# A. V. M. A.

**Published:** 1902-12

**Authors:** John J. Repp

**Affiliations:** Secretary


### A. V. M. A.

*To the Members of the American Veterinary Medical Association:*

THE next meeting of the American Veterinary Medical Association will be held at Ottawa, Canada, September 1, 2, 3, and 4, 1903. The vote of the Executive Committee was unanimous for that city as the next meeting-place. Invitations have been extended to us by the veterinarians of Ottawa and vicinity, by the Department of Agriculture of the Dominion, and by the civic authorities. Also Messrs. W. C. Edwards & Co., the senior member of which firm has been for many years a member of the Dominion Parliament and an extensive breeder of pure-bred live-stock, have invited the Association to visit their breeding establishment at Rockland, Ontario.

There is great enthusiasm among the veterinarians throughout the Dominion of Canada, and they are looking forward with great delight to this opportunity to entertain their professional brethren and their friends from the United States and Island possessions. Also veterinarians outside of the border of the Dominion are glad to have this opportunity to make the American Veterinary Medical Association a fact as well as a name, and anticipate great pleasure in visiting their sister nation upon the north and partaking of the far-famed hospitality and good cheer of its people.

The weather of Ottawa is especially delightful at the time of year when our meeting is to be held, and thus added pleasure will be given to the members and their friends in visiting the many places of interest for which Ottawa and its vicinity is noted.

Ottawa is easily accessible from all parts of the home of our organization by railroad and steamship lines. The trip will make as pleasant a vacation outing as could be devised.

It is expected that the Ottawa meeting can be made to far surpass any previous meeting, both in numbers in attendance and in new membership secured. The Association now numbers about 500. It ought to be 2000. It will soon reach that number if each member will exert his influence to accomplish it. It is urged upon each member that he will use his personal influence with veterinarians of his acquaintance who are eligible to membership to make application for such membership. Also, it is very much desired that each member who decides to attend the Ottawa meeting will, by personal interview and by letter, picture to his fellow members the pleasure and profit to be obtained from attendance at the meeting. If all will join in earnest effort to make the Ottawa meeting a grand success, the most sanguine will not be disappointed.

JOHN J. REPP,
Secretary.

